# CXCL12 and osteopontin from bone marrow-derived mesenchymal stromal cells improve muscle regeneration

**DOI:** 10.1038/s41598-017-02928-1

**Published:** 2017-06-12

**Authors:** Yasushi Maeda, Yasuhiro Yonemochi, Yuki Nakajyo, Hideaki Hidaka, Tokunori Ikeda, Yukio Ando

**Affiliations:** 1 Department of Neurology, National Hospital Organization Kumamoto Saishunso National Hospital, Kumamoto, Japan; 2 Department of Neurology, Graduate School of Medical Sciences, Kumamoto University, Kumamoto, Japan; 3 Department of Clinical Research Center, Faculty of Life Sciences, Kumamoto University, Kumamoto, Japan

## Abstract

Muscle satellite cells are essential for muscle regeneration. However, efficient regeneration does not occur without muscle-resident mesenchymal progenitor cells. We show here that bone marrow-derived mesenchymal stromal cells (Bm-MSCs) also facilitate muscle regeneration in Duchenne muscular dystrophy (DMD) model mice. Bm-MSCs transplanted into peritoneal cavities of DMD model mice with severe muscle degeneration strongly suppressed dystrophic pathology and improved death-related symptoms, which resulted in dramatic lifespan extension. Isolated single myofibers from Bm-MSC-transplanted mice manifested considerably less myofiber splitting compared with myofibers from non-transplanted mice, which indicated that transplantation significantly ameliorated abnormal regeneration. With regard to the number of satellite cells, several cells remained on myofibers from Bm-MSC-transplanted model mice, but satellite cells rarely occurred on myofibers from non-transplanted mice. Also, CXCL12 was crucial for muscle regeneration. CXCL12 facilitated muscle regeneration and paired box protein–7 (PAX7) expression after cardiotoxin-related muscle injury *in vivo*. The majority of primary muscle satellite cells sorted by integrin-α7 and CD34 expressed CXCR4, a receptor specific for CXCL12. CXCL12 strongly suppressed p-STAT3 expression in these sorted cells *in vitro*. CXCL12 may therefore influence muscle regeneration through STAT3 signaling in satellite cells. Targeting these proteins in or on muscle satellite cells may improve many degenerative muscle diseases.

## Introduction

Published studies revealed that many adult organs maintain regenerative competence because of various populations of resident stem/progenitor cells. In addition, another type of regeneration-facilitating cell has been found—mesenchymal progenitor cells (MPCs)—which secrete various soluble factors in damaged organs to provide an optimal regenerative milieu. However, detailed mechanisms of action of this regeneration remain poorly understood^1, 2^. In skeletal muscle, satellite cells are stem/progenitor cells, and their excellent regenerative ability approaches that of hematopoietic stem cells (HSCs). MPCs, however, reside in skeletal muscle, are PDGFR-α^+^, and differentiate into fibrogenic/adipogenic cells in response to cues from the surrounding microenvironment. Even during physical exercise, strongly contracted normal skeletal muscle is injured, and repair follows. Skeletal muscle therefore maintains a dynamic homeostasis between degeneration and regeneration. Regeneration, however, cannot compensate for pathological muscle degeneration, although increasing the population of satellite cells by means of regeneration-facilitating mesenchymal cells, if possible, may alleviate skeletal muscle symptoms.

MPCs occur in various tissues, e.g., adipose tissue^3^, skeletal muscle, bone marrow^3^, and umbilical cord^3^. Harvesting them from bone marrow is less invasive than harvesting them from skeletal muscle or adipose tissue. After MPCs are harvested, they can be easily expanded and give rise to many mesenchymal cells *in vitro*, enough for transplantation. We therefore harvested MPCs from mouse bone marrow, cultured them, and named them bone marrow-derived mesenchymal stromal cells (Bm-MSCs)^3^.

With regard to MSCs in bone marrow, nestin-expressing mesenchymal stem cells (nestin^+^ MSCs)^4^ and CXCL12-abundant reticular (CAR) cells^5, 6^ contribute to HSC niches. Nestin^+^ MSCs express higher levels of HSC maintenance factors, including CXCL12 (also named stromal cell-derived factor-1), osteopontin (OPN; also called secreted phosphoprotein-1, SPP-1), stem cell factor, and others. Although nestin^+^ MSCs do not seem to be identical to CAR cells, they doubtless have a close relationship^7^. In general, CXCL12-mediated activation of CXCR4 on HSCs is an indispensable signal for retention of HSCs in bone marrow.

As a more interesting finding, CXCL12 was reportedly important not only in HSC development^6, 8, 9^ but also in muscle development and regeneration. Because CXCL12 is highly expressed in injured muscle, we hypothesized that CXCL12 in muscle regeneration is important. CXCR4 is expressed on quiescent satellite cells^10^, but the biological effect of CXCL12 on satellite cells is still poorly understood.

To fully comprehend the molecular mechanisms of skeletal muscle regeneration, we must make intracellular and extracellular signals in muscle satellite cells obvious. STAT3 signaling was recently reported to regulate satellite cell function and skeletal muscle repair^11, 12^. STAT3 has been implicated in stem cell fate in several tissues, and STAT3 activation seems to reduce regeneration competence of satellite cells. In contrast, STAT3 inhibition promotes satellite cell expansion and enhances muscle repair. STAT3 may be thought of as a critical factor for satellite cells, whether immature or senile.

## Results

### Preparation and characterization of Bm-MSCs

We harvested mesenchymal cells from femoral and tibial bone marrow in *dystrophin/utrophin* double-knockout (dko) mice^13, 14^ by using Prockop’s protocol^3^. Bm-MSCs used in this experiment had only an *mdx* mutation in the *dystrophin* gene and a null mutation in the *utrophin* gene (Supplementary Fig. S1). We cultured these cells by using Prockop’s protocol^3^ (Supplementary Fig. S2a). We used a fluorescence-activated cell sorter (FACS) to identify cell surface markers in cultured cells. These cells were positive for stem cell antigen-1 (Sca-1), CD44, CD105, and CD106 and negative for CD45 and CD11b^15^ (Supplementary Fig. S2b). We confirmed their differentiation potentials via *in vitro* osteogenic and adipogenic differentiation^3^ (Supplementary Fig. S2c).

### Bm-MSC transplantation dramatically improved symptoms in dko mice *in vivo*

**Figure 1:**
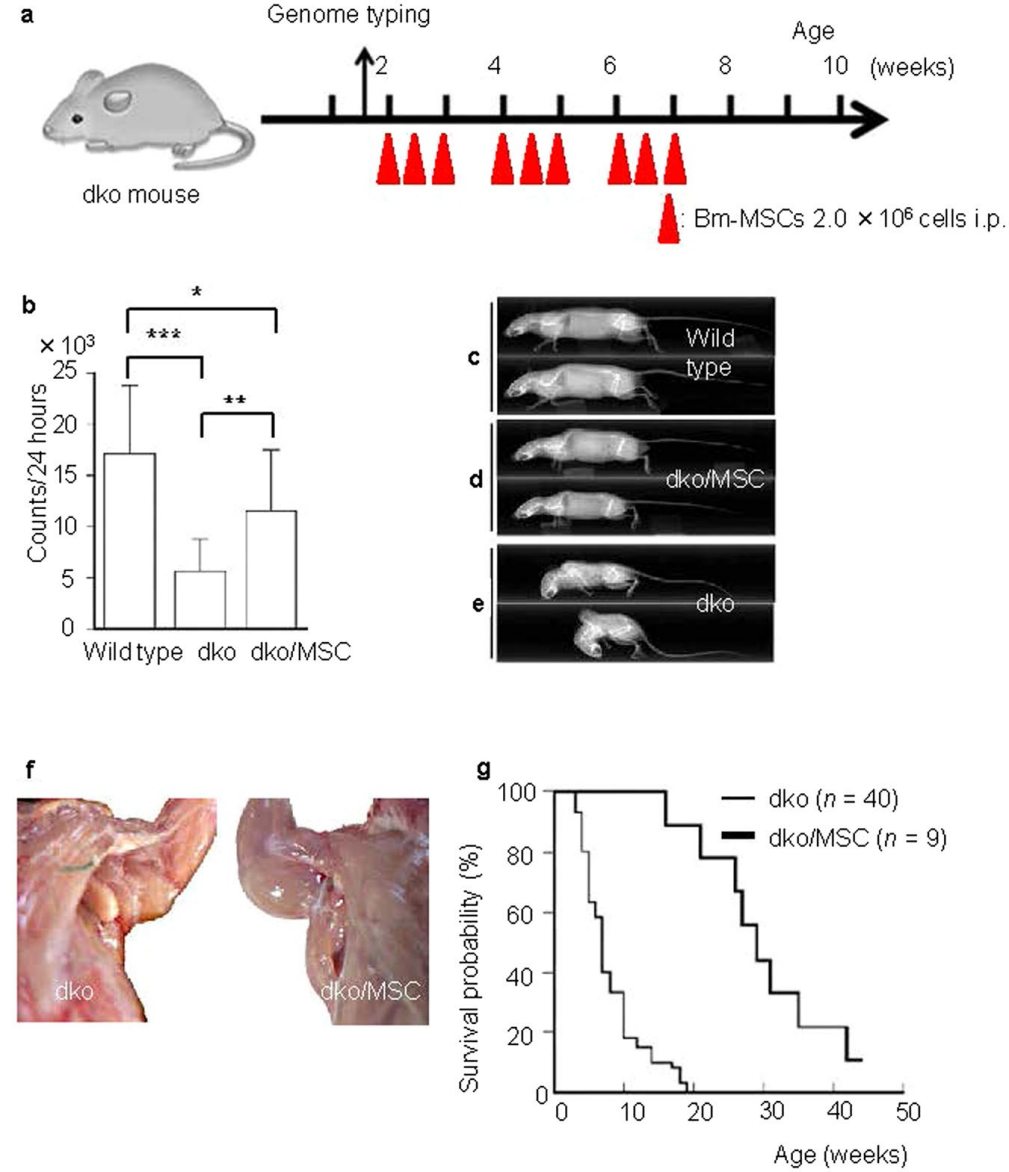
Bm-MSC transplantation dramatically improved symptoms in dko mice *in vivo*. (**a**) Transplantation protocol: Bm-MSCs harvested and cultured from dko mice were repeatedly transplanted into peritoneal cavities after genome typing. (**b**) After transplantation of the cells, locomotor activity improved at the age of 10 weeks. Locomotor activities during 24 hours (counts per 24 hours) for three groups were the following: wild type, 17,600 ± 7,800 (*n* = 9); dko, 5,400 ± 2,500 (*n* = 23); dko/MSC, 12,400 ± 7,900 (*n* = 9). *P < 0.05, **P < 0.01, ***P < 0.001. (**c**–**e**) X-ray studies showing that the build of Bm-MSC-transplanted dko mice (**d**) was indistinguishable from that of control wild-type mice (**c**) at 15 weeks of age. Without the Bm-MSC treatment, dko mice were quite small and had a severe skeletal deformity (kyphosis) (**e**). (**f**) Muscles in the trunk and forelimb. Bm-MSC treatment resulted in a larger muscle volume compared with the muscle volume of dko mice without treatment. (**g**) Transplantation improved longevity. The median survival of dko mice and dko/MSC mice was 9 weeks (*n* = 40) and 29 weeks (*n* = 9), respectively (*P* < 0.01).

To confirm that a secreted factor or factors affected myotube regeneration *in vivo*, we repeatedly transplanted (nine times) 2 million Bm-MSCs into the peritoneal cavities of dko mice^16^ between the second and seventh weeks after birth (Fig. 1a). Bm-MSCs had a s substantial effect *in vivo*: dko mice that had received the transplantation (dko/MSC) showed significantly improved locomotor activity for 24 hours at 10 weeks of age (p < 0.01) (Fig. 1b). At that age, dko mice rarely moved, and even when they attempted to move, they had a staggered gait. In contrast, dko/MSC mice moved actively (Supplementary movie). The appearance of dko/MSC mice also improved dramatically. Bm-MSC transplantation ameliorated the severe spinal curvature (kyphosis) and the small body size (Fig. 1c–f). Moreover, we were surprised at the increased longevity of mice after transplantation. No dko mouse survived longer than 20 weeks after birth^17^, but nearly all dko/MSC mice survived after 20 weeks, and some lived for 1 year (Fig. 1g). Because the transplantation treatments ended when mice were 7 weeks old in our protocol (Fig. 1a), we could not determine whether Bm-MSC transplantation would show greater effectiveness if continued.

### Bm-MSC transplantation improved myofiber histology and increased the number of satellite cells

**Figure 2:**
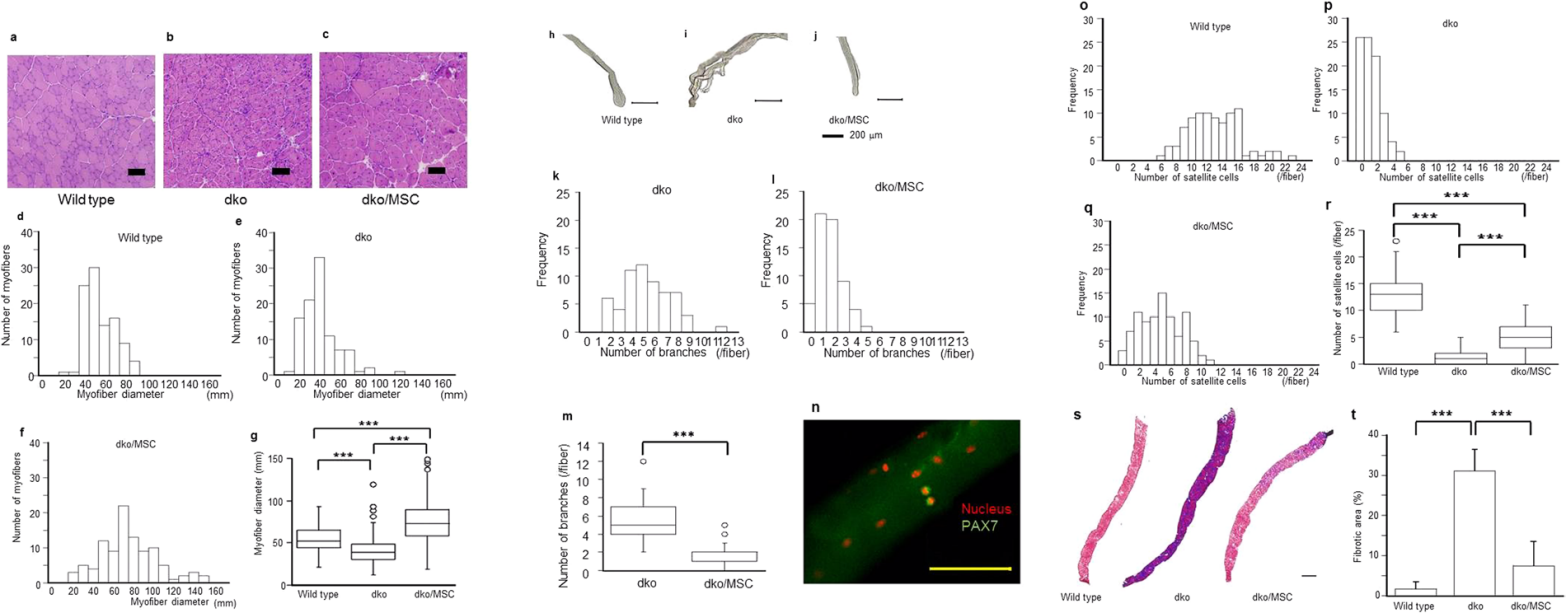
Bm-MSC transplantation improved myofiber histology and satellite cell numbers and reduced diaphragmatic fibrosis. (**a**–**c**) dko/MSC TA myofibers became hypertrophic when mice were 12 weeks old, but central nuclei were retained. Scale bars: 50 μm. (**d–g**) Mean myofiber diameters for wild-type, dko, and dko/MSC mice were 55.5 ± 14.7 μm, 41.4 ± 18.1 μm, 74.9 ± 28.2 μm, respectively (*n* = 100 in each group; ***P < 0.001). (**h–j**) Isolated calf myofibers from the three groups had completely different appearances. The dko myofibers had many branches, but Bm-MSC transplantation suppressed branching. (**k–m**) The dko myofibers had 5.4 ± 2.1 branches, versus 1.8 ± 1.1 for the dko/MSC myofibers (*n* = 60; ***P < 0.001). (**n**) A representative immunofluorescence image (nucleus, red; PAX7, green). Immunofluorescence allowed the identification of satellite cells on an isolated myofiber. Scale bar: 100 μm. (**o–r**) Bm-MSC transplantation affected satellite cell number. Satellite cells rarely occurred on isolated dko myofibers; Bm-MSC transplantation, however, significantly increased the number of cells: 13.0 ± 3.4, 1.4 ± 1.3, and 4.9 ± 2.7 for wild-type, dko, and dko/MSC mice, respectively (*n* = 90 in each group; ***P < 0.001). (**s**) Bm-MSC transplantation suppressed fibrotic degeneration. Masson’s trichrome staining (with aniline blue) revealed interstitial fibrosis in transverse muscle sections from 12-week-old mice. Scale bar: 500 μm. (**t**) The areas of fibrosis in dko/MSC mice decreased significantly compared with such areas in dko mice: wild-type, dko, and dko/MSC fibrotic areas (%) were 1.8 ± 1.7, 31.2 ± 5.3, and 7.5 ± 6.0, respectively (*n* = 4 in each group; ***P < 0.001).

Histological analysis revealed that skeletal muscle fibers of dko/MSC mice were hypertrophic compared with muscle fibers of wild-type mice, but central nuclei remained (Fig. 2a–g). Because the muscle fibers of age-matched dko mice were quite small in diameter, the effect of Bm-MSCs involved something other than normalizing fiber size. Other studies reported that the *mdx* mouse, another DMD model mouse that carries a splicing mutation in the *dystrophin* gene and manifests hypertrophic myofibers with central nuclei, was indistinguishable from the wild-type mouse in terms of lifespan and locomotor activity^18, 19^. Our study indicated that, similar to the situation with the *mdx* mouse, hypertrophic myofibers of the dko/MSC mice that the Bm-MSCs produced may be responsible for these effects. We therefore analyzed isolated single myofibers from gastrocnemius and soleus muscles. Inasmuch as we achieved similar results when we isolated single muscle fibers by the methods mentioned, we concluded that Bm-MSC cell transplantation resulted in different branching characteristics of isolated single muscle fibers obtained from wild-type mice compared with fibers obtained from dko/MSC mice. Single myofibers from wild-type mice had no branches and dko single myofibers had many branches. Single myofibers from dko/MSC mice, however, had fewer branches than did myofibers from dko mice (Fig. 2h–m). Because the many branches of the single dko myofibers tangled easily and were vulnerable to mechanical stress, isolation of a single myofiber was difficult (Fig. 2h–m). The same sort of vulnerability may occur *in vivo*. Bm-MSC transplantation suppressed myofiber branching, which indicated incomplete regeneration and strongly suggested that Bm-MSCs affect regeneration, that is, muscle satellite cell functions.

In addition, we compared the number of satellite cells on single myofibers isolated from age-matched wild-type, dko, and dko/MSC mice. The dko single myofibers had fewer satellite cells than did wild-type myofibers, but Bm-MSCs caused an increased number of satellite cells on dko/MSC myofibers compared with dko myofibers (Fig. 2n–r).

### Bm-MSC transplantation suppressed fibrosis in diaphragms

Both *mdx* and wild-type mice have certain similar features, but only *mdx* mice manifest a continuously fibrotic diaphragm^16^. To determine the effect of Bm-MSCs on fibrosis, we compared the diaphragms of dko and dko/MSC mice. Masson’s trichrome staining revealed strong inhibition of fibrosis by Bm-MSC transplantation (Fig. 2s,t). This suppression may reflect immunomodulation by Bm-MSCs^16^.

### Bm-MSCs improved myotubes *in vitro*

**Figure 3:**
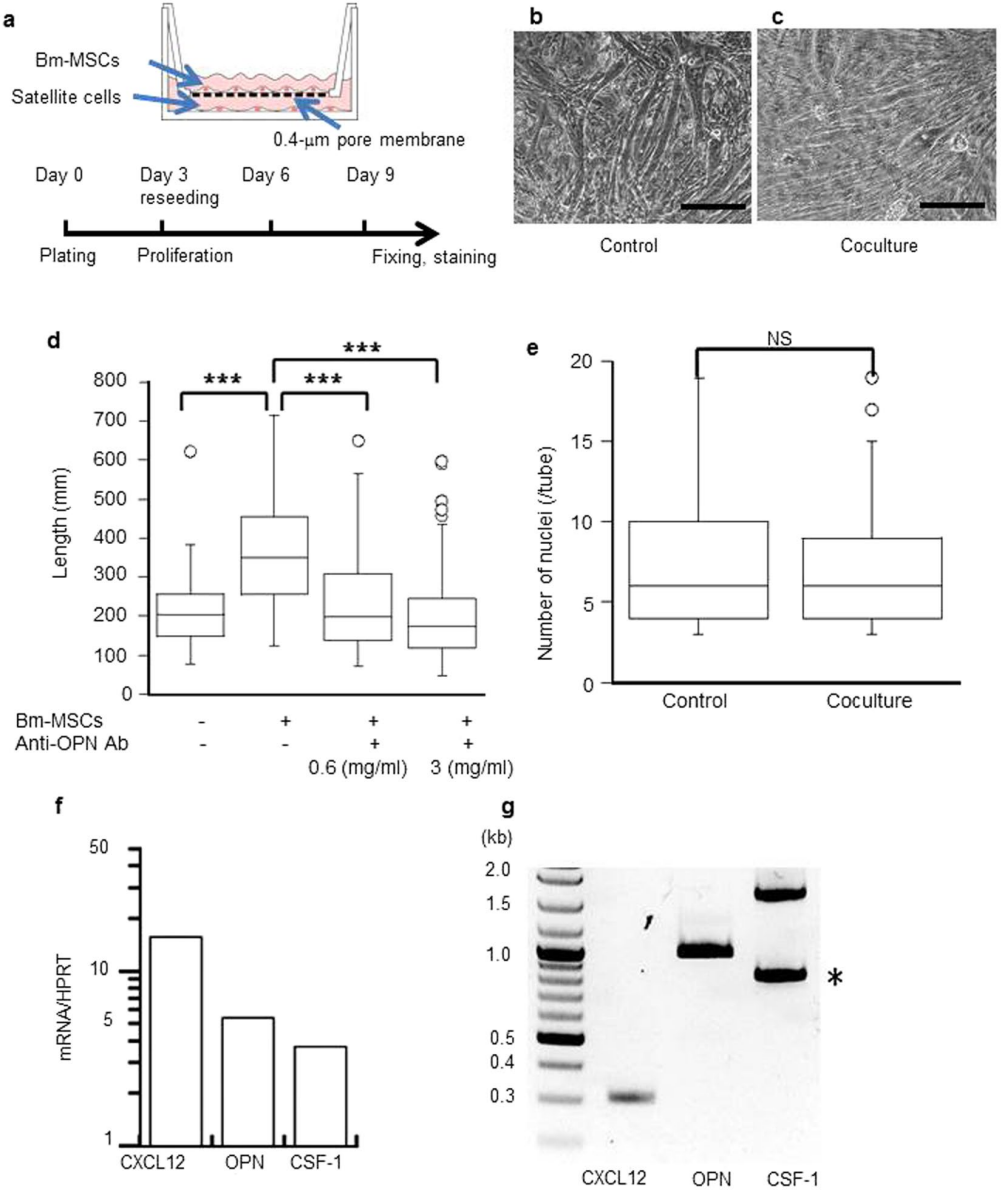
Bm-MSCs produced various growth factors and affected *in vitro* myotube formation. (**a**) Bm-MSCs (1.0 × 10^5^ cells per insert) were added to culture inserts 1 day before starting cocultures with single myofibers isolated from the calf muscle of the wild-type mouse. The culture design is described in detail in Material Methods. Micrographs showing that, compared with the control culture (**b**), myotubes cocultured with Bm-MSCs (**c**) were longer and grew in a definite direction. Scale bars: 100 μm. (**d**) Addition of anti-OPN antibody to the coculture inhibited these effects in an antibody concentration-dependent manner. Myotube length: control, 216 ± 93.3 μm; coculture, 369.7 ± 174.5 μm (***P < 0.001; *n* = 65–75 in each group). Data represent at least three independent experiments. (**e**) The number of nuclei in each myotube in the control and coculture groups did not differ. This result suggests that the fusion frequency of the myoblasts was the same. NS, not significant. (**f**) The mRNA-PCR result showing that Bm-MSCs expressed high amounts of CXCL12, OPN, and CSF-1. HPRT, hypoxanthine-guanine phosphoribosyltransferase. (**g**) RT-PCR showing full-length CXCL12, OPN, and CSF-1 in Bm-MSCs. *Indicates a fragmentation of OPN.

As recent reports showed, factors secreted from MSCs are quite important for tissue regeneration and remodeling^20, 21^. To evaluate the effect of our Bm-MSCs on muscle regeneration, we cocultured satellite cells from single myofibers of gastrocnemius and soleus muscles obtained from wild-type mice with dko Bm-MSCs (Fig. 3a). Because newly formed myotubes matured enough to contract spontaneously after 12 days of culture, we performed analyses on the ninth day after the start of culture. Bm-MSCs clearly affected myotube morphology: these myotubes were longer than control myotubes (Fig. 3b,c). Such facilitation was attenuated by addition of neutralizing antibody against OPN to the coculture (Fig. 3d). Moreover, the two groups had the same number of nuclei in each myotube, which indicated that the morphological difference between the groups did not arise from the frequency of myoblast fusion (Fig. 3e).

### Bm-MSCs produced various growth factors

Bm-MSCs were placed in culture inserts to prevent direct contact with single fibers, so that myotube formation was affected by only secreted molecules. We quantified the mRNAs in Bm-MSCs, with a focus on secreted growth factors, by using a reverse transcription (RT)-PCR array. CXCL12, OPN, and CSF-1 were highly expressed (Fig. 3f,g). In Bm-MSC culture medium, we detected the CXCL12 protein by using an enzyme-linked immunosorbent assay (Supplementary Fig. S3). CXCL12 is a typical bone marrow-derived chemokine that maintains stemness of HSCs via CXCR4^22, 23^. Extremely high expression of CXCL12 and successful osteogenesis/adipogenesis of our Bm-MSCs suggested that these cells may be derived from reticular cells with abundant CXCL12 in bone marrow^6^.

### CXCL12 improved myofiber histology and increased PAX7 *in vivo*

**Figure 4:**
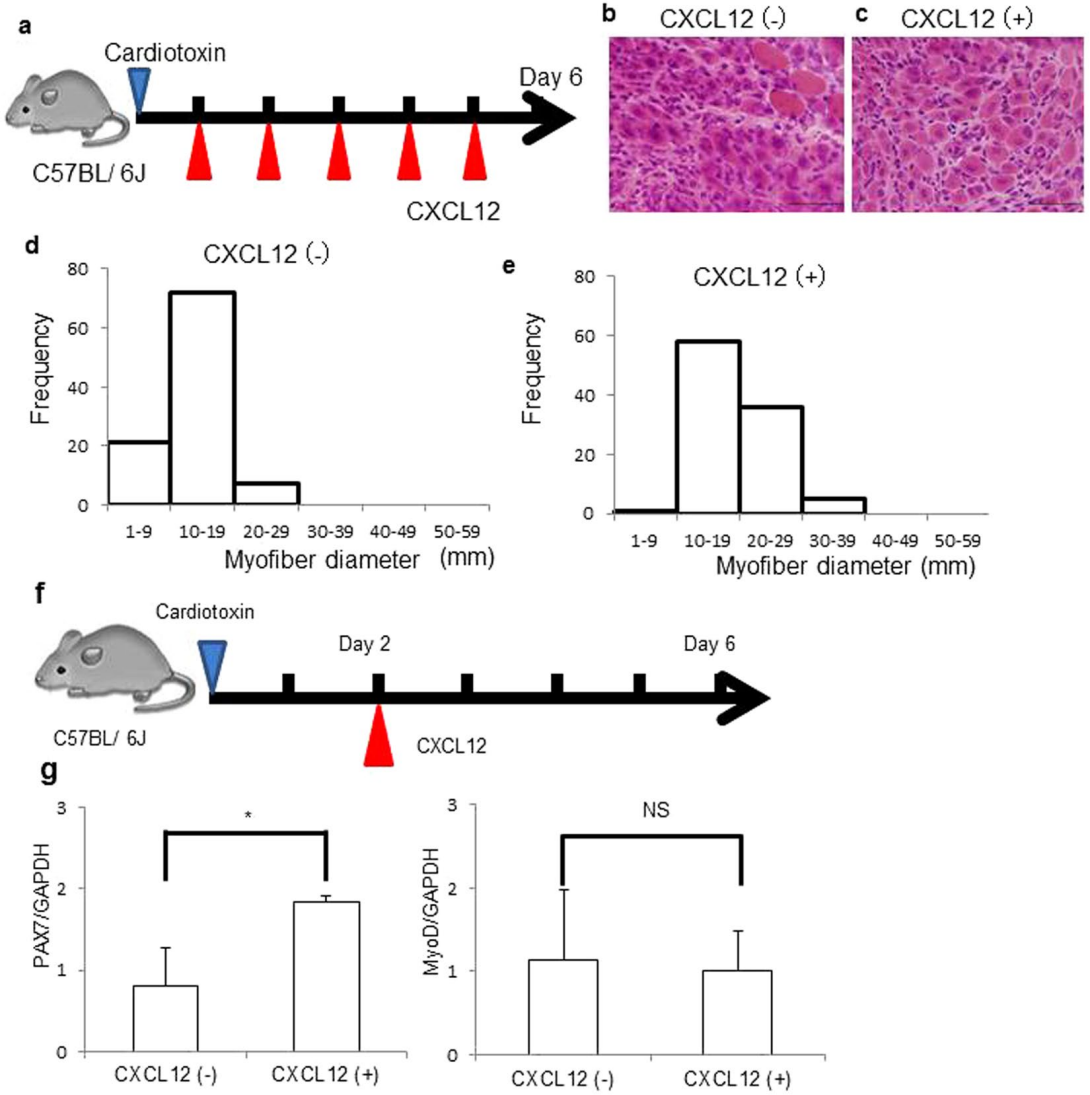
CXCL12 improved myofiber histology and increased PAX7 mRNA *in vivo*. (**a**) We injected 15 μg of CTX (L8102; LATOXAN SAS) into a right TA muscle in six 10-week-old male C57BL/6 mice. We also repeatedly injected CXCL12 into the peritoneal cavities of three of these mice for 5 days. We killed these mice on day 6 and analyzed the muscles. (**b**-**e**) TA myofibers became hypertrophic in mice that received CXCL12 injections. Scale bars: 100 μm. (**f**) We injected 15 μg of CTX into a right TA muscle in six 10-week-old male C57BL/6 mice. On day 2, we injected CXCL12 into the peritoneal cavities of three of these mice. We killed these mice on day 6 and analyzed the muscles. (**g**) PAX7 mRNA increased but MyoD was unchanged: PAX7/GAPDH: 0.81 ± 0.47 and 1.84 ± 0.08 for the control (no CXCL12) and CXCL12 groups, respectively (*n* = 3 in each group; *P < 0.05). MyoD/GAPDH: 1.13 ± 0.84 and 1.01 ± 0.47 for the same groups, respectively (*n* = 3 in each group).

To evaluate the effects of CXCL12 on muscle regeneration and satellite cells, we injected CXCL12 into the peritoneal cavities of wild-type mice posterior to a cardiotoxin (CTX) injection into skeletal muscle that was administered to induce muscle regeneration (Fig. 4a). Histological analysis revealed that skeletal muscle fiber diameters increased in wild-type mice injected with CXCL12 compared with mice receiving no CXCL12 injections (Fig. 4b–e). We next quantified the mRNAs of the muscle regeneration factors PAX7 and myogenic differentiation-1 (MyoD) in tibialis anterior (TA) muscles by using RT-PCR (Fig. 4f,g). MyoD mRNA did not increase but PAX7 mRNA did.

### CXCL12 completely halted p-STAT3 expression

**Figure 5:**
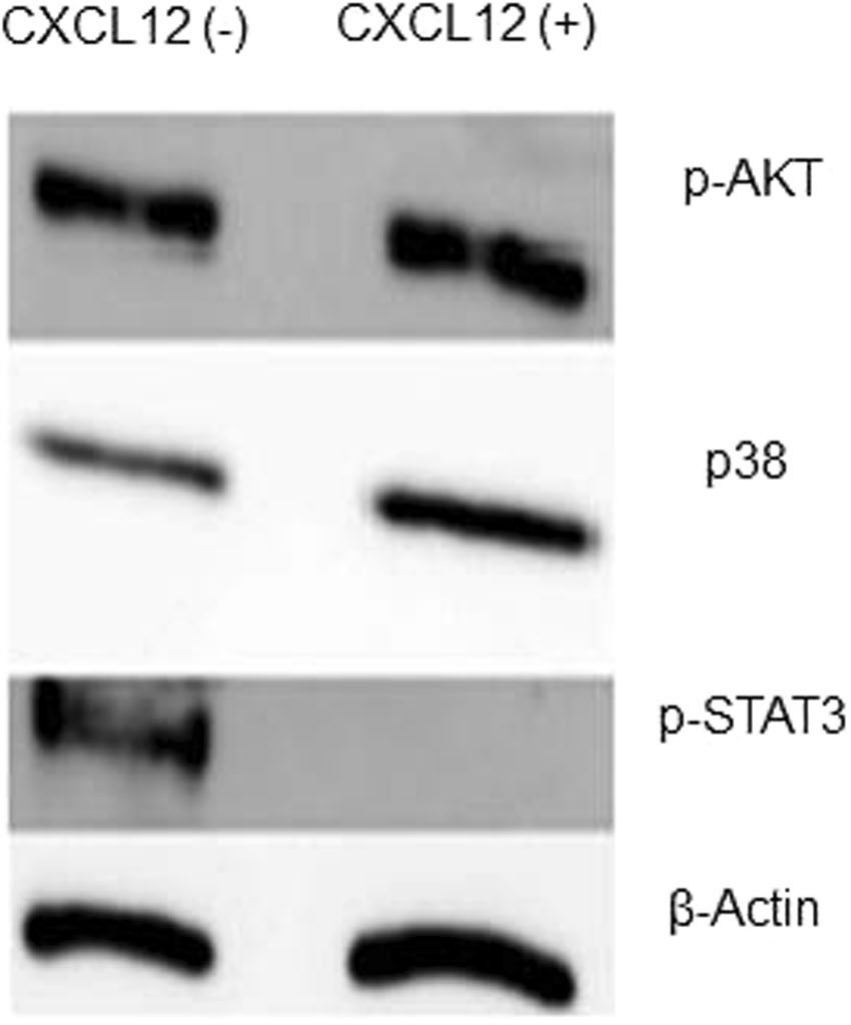
Western blot showing that CXCL12 completely suppressed p-STAT3 expression in satellite cells, but p-AKT and p38 were unchanged.

Because CXCL12 increased PAX7 mRNA and promoted muscle regeneration *in vivo*, we investigated the intracellular signaling pathway *in vitro*. We cultured muscle satellite cells with CXCL12 and analyzed proteins by using Western blotting (Fig. 5). CXCL12 completely suppressed p-STAT3 expression in satellite cells. Also, we should note that inhibition of the phosphorylation of STAT3 by CXCL12 is a satellite cell-specific phenomenon, because we could not produce the same results in other cell lines (Supplementary Fig. S4).

## Discussion

We initiated this study on the basis of the hypothesis that mesenchymal cells could affect skeletal muscle regeneration. Skeletal muscle develops in the epaxial and hypaxial domains of a dermomyotome from mesodermal mesenchymal cells during ontogeny. As the skeletal muscle develops, the PAX3^+^ and PAX7^+^ cells, which become muscle satellite cells in postnatal muscle, remain quiescent before muscle degradation and regeneration^22–24^. That is, both embryonic muscle cells and adult muscle satellite cells originate from mesenchymal cells. Besides this similarity, myoblasts derived from quiescent satellite cells during muscle regeneration resemble embryonic myogenic cells in terms of the gene expression profile. As satellite cells are activated to repair injured muscle fibers, PAX7, Myf5, MyoD, and then myogenin are expressed in a temporal sequence^25^. These factors are also expressed in embryonic muscle progenitor cells. All this evidence demonstrates that what happens in an embryo also occurs during muscle regeneration. Hence, we expected that mesenchymal cells in adult skeletal muscle could influence satellite cell activation.

Some groups have identified skeletal muscle-resident MPCs in mice^1, 2, 26^. These cells are non-myogenic, interstitial cells in skeletal muscle that are characterized by the absence of satellite cell surface markers and by the expression of PDGFR-α^2^ or Sca-1^1^. These cells possess dual and contrasting features, such as functional support for satellite cells in muscle regeneration, and being a source of ectopic fat deposition and fibrosis. Elucidating how MPCs choose which features they manifest is critical. On the basis of our data from studies of Bm-MSC transplantation in the DMD model mouse—results including hypertrophic regenerated muscle fibers, many residual satellite cells, and effective prevention of fibrosis of the diaphragm in Bm-MSC-transplanted mice—our transplantation method may help determine the fate of the muscle-resident MPCs, as they choose to support satellite cells in muscle regeneration but are also a source of ectopic fat deposition and fibrosis.

In our experiments with the DMD model mouse, we repeatedly transplanted Bm-MSCs instead of muscle-resident MPCs into peritoneal cavities. At first, we transplanted genetically marked Bm-MSCs to help with their identification, but inasmuch as the transplanted cells were not detected in mice 7 days after transplantation, the dramatic effects observed in the Bm-MSC-transplanted mice must depend on a factor or factors derived from the Bm-MSCs. Such a factor or factors from these Bm-MSCs must support the satellite cells, via the systemic circulation, and affect what occurs between regenerating satellite cells and MPCs. Of note, in our experiments we induced the mesenchymal cells from bone marrow to modify, at a distance, what was happening at sites where muscle satellite cells and muscle-resident MPCs interacted during regeneration. That is, we observed the effects of bone marrow on muscle regeneration.

With regard to factors that circulate systemically that affect adult stem cells, we are reminded of the heterochronic parabiosis mouse model in which a young mouse and an aged mouse shared the circulatory system, and the aged satellite cells were rejuvenated by means of exposure to the circulating factor or factors from the young mouse though the shared circulation^27^. A recent study showed the importance of the systemic concentration of circulating oxytocin for satellite cell activity^28^. One or some secreted factors from our Bm-MSCs may thus serve as the circulating factors for satellite cell activity. Our Bm-MSCs produced various growth factors including CXCL12, OPN, and CSF-1. CXCL12, also called stromal cell-derived factor-1, is a typical bone marrow-derived chemokine. High expression of CXCL12 and successful osteogenesis/adipogenesis of our Bm-MSCs suggested that these cells may derive from CAR cells^5, 6^ or nestin^+^ MSCs in bone marrow^4^.

OPN is a multifunctional molecule and has pivotal roles in inflammation, bone formation, and tissue repair and remodeling. In studies with C2C12 myoblasts and fetal myoblasts, OPN was important primarily during early phases of myogenesis, when it aided fusion and differentiation of myoblasts^29^. Indeed, we confirmed that OPN derived from Bm-MSCs facilitated fusion and differentiation of the primarily isolated myoblasts *in vitro* and that such facilitation was attenuated by adding neutralizing antibody against OPN (Fig. 3d). Also, because the literature suggests the involvement of OPN in HSC retention and maintenance, we should investigate the effects of OPN in terms of muscle satellite cell maintenance.

We here discussed mainly the indirect supporting effects on satellite cells of Bm-MSC transplantation, such as the regenerative environment related to muscle-resident MPCs, but in the future we should also address the direct supporting effects of Bm-MSC transplantation on satellite cells.

CXCL12 plays a role in diverse cellular functions, including embryogenesis, immune surveillance, response to inflammation, homeostasis in tissues, and tumor growth and metastasis^5, 6, 8, 9^. CXCL12 is the key molecule that maintains the stemness of HSCs via the CXCL12-specific receptor CXCR4, which is expressed on the cell surface of HSCs. With regard to skeletal muscle, dormant satellite cells express CXCR4^30^ on their surfaces, and CXCL12 is highly expressed in regenerating muscle after injury. Our primary mouse satellite cells that we harvested by using FACS with a combination of cell surface markers (including integrin-α7^+^ and CD34^+^) successfully differentiated into myotubes in culture (Supplementary Fig. S5a,c). Immediately after the isolation, we also detected CXCR4 expression, by using FACS, in a majority of the cells (Supplementary Fig. S5a). Downregulation of CXCR4 expression after satellite cell activation seems contradictory to the high CXCL12 concentration in response to muscle injury. However, the idea that a high concentration of CXCL12 is necessary so that some satellite cells continue to undergo self-renewal is attractive. In agreement with this idea, our experiment in which we administered CXCL12 before muscle destruction with CTX showed regeneration accompanying significantly higher PAX7 induction than that during usual regeneration without CXCL12 administration in wild-type mice (p < 0.05). We also compared MyoD expression in two groups—with and without CXCL12—and confirmed equally high MyoD induction. Other groups reported that CXCL12 improved migration of cells that aid muscle regeneration, with the result being that muscle regeneration improved^31–33^. The possibility exists that CXCL12 may maintain PAX7 expression in satellite cells. This mechanism is quite important for muscles to maintain regenerative competence through a lifetime. Senile muscle, however, does not possess vital regenerative ability^27, 34–38^. Aging is associated with a diminished regenerative ability of the muscles and a loss of muscle volume (sarcopenia). In rodents, the senile muscle and satellite cell environment disrupted satellite cell function and muscle regenerative ability. Intracellular signaling in satellite cells in aged muscle is greatly altered compared with cell signaling in young muscle. Constitutive activation of p38 kinase^39^ and STAT3 and reduction of Notch signaling have been observed in aged satellite cells^38^. In this regard, we found an intriguing event induced in satellite cells cultured with CXCL12: CXCL12 abolished STAT3 signaling.

STAT3 is a latent transcription factor that mediates extracellular signals such as cytokines and growth factors via interaction with polypeptide receptors at the cell surface^40^. The inflammatory cytokine interleukin-6 and the growth factor epidermal growth factor are well-known extracellular factors that activate STAT3 signaling. After STAT3 protein becomes activated, primarily by tyrosine phosphorylation, the activated protein (p-STAT3) translocates to the nucleus and binds to sequence-specific DNA elements for transcription of target genes^41^. An interesting finding is that one of the p-STAT3 target genes—*MyoD*—is a master gene for skeletal muscle differentiation, which means that activation of STAT3 signaling drives quiescent satellite cells to proliferate and differentiate into myoblasts and muscle fibers. Certain groups recently showed that STAT3 signaling in aged satellite cells was constitutively activated by long-term interleukin-6 stimulation, which led to a loss of regenerative capacity^11, 12^. It is critical for satellite cells to maintain regenerative ability whether STAT3 signaling is active or not. In fact, we found suppressed STAT3 signaling, rather than activated STAT3 signaling, in satellite cells with CXCL12 *in vitro*. We used an anti-p-STAT3 antibody that recognized phosphorylation of a single tyrosine residue, Tyr705, which is typically phosphorylated by receptor tyrosine kinases such as EGFR, KDR, and MET or by non-receptor tyrosine kinases such as JAKs^42, 43^. In our study, we did not detect p-STAT3 in cultured satellite cells with CXCL12 by Western blotting, whereas according to the protein array studies the STAT3 protein content did not differ whether the satellite cells were cultured with CXCL12 or not (Supplementary Table S1). That is, CXCL12 indirectly inhibited the phosphorylation of the Tyr705 of STAT3 in muscle satellite cells.

In summary, our data presented here provide important information that is useful for treating various degenerative muscle diseases including muscular dystrophy and locomotive syndrome. Mesenchymal cells, CXCL12, and chemicals targeting CXCR4 and STAT3 may be promising approaches to use in strategies to preserve an effective regenerative competence of satellite cells.

## Materials and Methods

### Mice

All animals were maintained under conditions of a 12-hour light-dark cycle (light from 07:00 to 19:00) at 22 ± 1 °C and *ad libitum* food and water. The Animal Care and Use Committee of Kumamoto University School of Medicine approved the protocols for the animal experiments. We used dko mice, C57BL/10 mice, and C57BL/6 mice (Central Institute for Experimental Animals, Kawasaki, Japan). Mice were housed in the Center for Animal Resources and Development of Kumamoto University. The dko mice used here were originally generated by Deconinck and colleagues^13^. We obtained experimental dko mice by crossing *utrophin* heterozygous mice onto an *mdx* background. We genotyped DNA obtained by means of a tail biopsy of 2-week-old mice via PCR with three primers, as reported by Deconinck and colleagues^13^. We identified the point mutation in the *dystrophin* gene in the *mdx* mouse by combining two sets of PCRs: a wild-type-specific primer pair (forward primer: 5′-AACTCATCAAATATGCGTGTTAGTG-3′, reverse primer: 5′-GTCACTCAGATAGTTGAAGCCATTTAG-3) and a mutation-specific primer pair (forward primer: 5′-AACTCATCAAATATGCGTGTTAGTG-3′, reverse primer: 5′-GTCACTCAGATAGTTGAAGCCATTTAT-3′)^44^. PCR analysis to determine *utrophin* knockout status used a forward primer complementary to exon 7 of mouse *utrophin* (5′-GTGAAGGATGTCATGAAAG-3′) and reverse primers complementary to either intron 7 (5′-TGAAGTCCGAAAGAGATACC-3′) or the phosphoglycerate kinase promoter located within the neo knockout cassette (5′-ACGAGACTAGTGAGACGTGC-3′). Reactions were performed with genomic DNA for 35 cycles under the following conditions: 94 °C, 30 seconds; 57 °C, 30 seconds; and 72 °C, 25 seconds.

### Bm-MSC transplantation

Cultured Bm-MSCs were washed with PBS and lifted by incubation with trypsin/EDTA for 2 minutes at 37 °C. Trypsin was quenched by adding CEM, and cells were centrifuged and washed twice with PBS to remove serum. Cells were resuspended in PBS, and then 2.0 × 10^6^ cells per mouse were injected into the peritoneal cavity of a dko mouse. According to the injection schedule shown in Fig. 2, before the Bm-MSC injection, genome typing PCR was performed for the *utrophin* gene to select the dko mouse. The ninth injection was given at the end of the seventh week after birth.

### Motor performance and survival analysis

Locomotor activity was analyzed with nine 12-week-old C57BL/10 male mice and thirty-two 12-week-old dko male mice. To quantify locomotor activity, we used an automated electronic activity counter (NS-AS01; NeuroScience, Tokyo, Japan). Each mouse was put into a clear acrylic cage (24 cm × 17 cm × 12 cm), and its activity was measured for 24 hours by using the activity counter placed 15 cm above the cage. All mice were housed in rooms under conditions of a controlled temperature of 22 ± 2 °C, relative humidity of 50 ± 10%, and 12-hour light-dark cycle. Survival of 49 male dko mice was analyzed by using Kaplan-Meier analysis, with comparisons made by means of the log-rank test.

### Histopathological analysis and immunostaining of the TA muscle and diaphragms

After mice were killed by cervical dislocation, TA muscles and diaphragms were removed. The TA muscles were quickly frozen in isopentane precooled with liquid nitrogen. Diaphragms were embedded in optimal cutting temperature compound (Sakura Fine Technical, Tokyo, Japan) once and then frozen in precooled isopentane. The frozen TAs and diaphragms were sectioned at a 10-μm thickness and stained with hematoxylin and eosin or Masson’s trichrome. TA muscle fiber diameters were measured by using a measurement module installed on the All-in-One Fluorescence Microscope BZ-9000 (Keyence, Osaka, Japan). Diaphragmatic fibrosis was evaluated by using Masson’s trichrome staining. The area of blue staining indicating fibrosis was calculated from the cross-sectional area (%) of the entire diaphragm by using WinROOF software (version 5.6; Mitani, Fukui, Japan) with an optical microscope (DP70-WPCXP; Olympus, Tokyo, Japan). To stain muscle satellite cells, we used anti-PAX7 (clone PAX7, catalog MAB1675; R&D Systems, Minneapolis, MN). Goat Anti-Mouse IgG H&L (Alexa Fluor 488) (catalog ab150113; Abcam plc, Cambridge, UK) was used as the secondary antibody. We used DAPI (catalog D1306; Thermo Fisher Scientific, Waltham, MA) to stain nuclei. For the immunostaining studies, we used the All-in-One Fluorescence Microscope BZ-9000 (Keyence).

### Real-time RT-PCR array of growth-related factors expressed by Bm-MSCs

We analyzed which cell growth-related factors the Bm-MSCs expressed by using a real-time RT-PCR array. For this purpose, we chose the RT^2^ Profiler PCR Array (QIAGEN, Valencia, CA) and followed the manufacturer’s instructions. Total RNA isolated from 1.0 × 10^7^ Bm-MSCs by means of TRIzol Reagent (Thermo Fisher Scientific) was further purified by using the miRNeasy Mini Kit (QIAGEN) to remove DNA contamination. The purity of this RNA was guaranteed by using two methods. First, the concentration and purity of RNA were determined by measuring absorbance in a NanoDrop spectrophotometer (Thermo Fisher Scientific). Second, an aliquot of RNA was run on the Agilent Bioanalyzer with an RNA 6000 Nano LabChip. We verified the presence of two sharp peaks for both 18S and 28S rRNAs. We chose RNA samples satisfying the manufacturer’s criteria for high-quality RNA for first-strand synthesis. We synthesized first-strand DNA from 0.5 μg of total RNA by using the RT^2^ First Strand Kit (QIAGEN). Finally, we performed real-time PCR by using the RT^2^ Profiler PCR Array, according to the manufacturer’s instructions, in combination with RT^2^ SYBR Green Mastermix (QIAGEN) on a model 7000 cycler (Applied Biosystems, Carlsbad, CA). We analyzed the data via the PCR Array Data Analysis Web Portal at www.SABiosciences.com/pcrarraydataanalysis.php. Briefly, after the real-time PCR, we normalized the original expression level of each gene of interest to the expression level of a housekeeping gene, that is, we divided the expression levels of the two genes. We then compared the expression level of the gene of interest with that of the housekeeping gene.

### CXCL12 injection into peritoneal cavities and histopathological analysis

We injected 15 μg of CTX (L8102; LATOXAN SAS, Valence, France) into a right TA muscle in six 10-week-old male C57BL/6 mice. CXCL12 was repeatedly injected into the peritoneal cavities of three of these mice for 5 days. We killed these mice on day 6 by using cervical dislocation and analyzed the TA muscles. Frozen TA muscles were sectioned 10 μm thick and were stained with hematoxylin and eosin or Masson’s trichrome. TA muscle fiber diameters were measured by using a measurement module installed on the All-in-One Fluorescence Microscope BZ-9000 (Keyence).

### CXCL12 injection into the peritoneal cavities and RT-PCR

We injected 15 μg of CTX into a right TA muscle in six 10-week-old male C57BL/6 mice. CXCL12 was injected into the peritoneal cavities of three of these mice on day 2. We killed these mice on day 6 by using cervical dislocation and removed the TA muscles by microdissection. We homogenized these muscles and isolated RNA from these samples by using the RNeasy Mini Kit (QIAGEN). We achieved total RNA purification and first-strand cDNA synthesis. Amplification of PAX7, MyoD, and GAPDH by PCR was performed by using Ampdirect Plus (Shimazu, Kyoto, Japan) and NovaTaq DNA Polymerase (Novagen, Darmstadt, Germany) according to the manufacturers’ instructions. Primer sequences were as follows: PAX7 forward primer: 5′-CCGTGTTTCTCATGGTTGTG-3′, reverse primer: 5′-GAGCACTCGGCTAATCGAAAC-3′. MyoD forward primer: 5′-AGCACTACAGTGGCGACTCA-3′, reverse primer: 5′-GCTCCACTATGCTGGACAGG-3′. GAPDH forward primer: 5′-TGATGACATCAAGAAGGTGGTGAAG-3′, reverse primer: 5′-TCCTTGGAGGCCATGTAGGCCAT-3′.

### Western blotting of satellite cells

We isolated satellite cells by using FACS, harvested 1.0 × 10^5^ cells, and plated them in 6-well tissue culture plates with 50% Matrigel (Becton, Dickinson, Franklin Lakes, NJ). We cultured the cells in medium that consisted of Dulbecco modified Eagle’s medium plus GlutaMAX-I (Gibco, Grand Island, NY) supplemented with 2 mM l-glutamine, 10% horse serum, and 20% FBS and incubated them in a 5% CO_2_ incubator at 37 °C for 6 days. We changed this medium and added CXCL12 every 5 days. On day 6, we performed Western blotting. We collected proteins with 8 M urea (100 mM Na_3_PO_4_, 10 mM Tris-HCl, pH 8.0). Samples were homogenized and centrifuged (20 minutes, 13,200 rpm), and the supernatant was collected. Protein concentration was measured by using the BCA Protein Assay Kit (Pierce Chemical, Rockford, IL). Equal amounts of protein were heat-denatured in sample-loading buffer (Bio-Rad Laboratories, Hercules, CA) at 85 °C for 5 minutes. Samples were resolved by means of SDS-PAGE and transferred to nitrocellulose membranes with the iBlot Gel Transfer Device (Invitrogen, Carlsbad, CA). The filters were blocked with Tris-buffered saline containing 0.05% Tween and 5% nonfat dry milk and incubated overnight at 4 °C with the following antibodies: anti-p38 (catalog 8690S; Cell Signaling Technology), anti-p-STAT3 (catalog 9145S; Cell Signaling Technology), anti-p-p44/42 (catalog 4370S; Cell Signaling Technology), anti-p-AKT (catalog 4060S; Cell Signaling Technology), and anti-β-actin (clone AC-74, catalog A5316; Sigma, St. Louis, MO). Polyclonal Goat Anti-rabbit Immunoglobulins/horseradish peroxidase (catalog P0448; Dako, Glostrup, Denmark) and Polyclonal Rabbit Anti-mouse Immunoglobulins/horseradish peroxidase (catalog P0447; Dako) were used as secondary antibodies. Membrane-bound immune complexes were detected by means of the ECL Prime Western Blotting Detection System (GE Healthcare, Pittsburgh, PA) and LAS-4000 mini EPUV (Fujifilm, Tokyo, Japan). Densitometric analysis via ImageJ software allowed quantification of the bands.

### Statistics

We performed all studies with at least three different cultures or animals in independent experiments. Data are expressed as means ± SD. We determined statistical differences by means of Student’s *t* test or one-way analysis of variance, with differences among groups being analyzed via a Tukey-Kramer post-hoc analysis. Statistical differences in survival were assessed by using the log-rank test.

## Electronic supplementary material

suplementary methods, suplementary figure legend

suplementary figure S1–S5

suplemental table S1

dko/MSC mice
