# Supplementary material for: CXCL12 and osteopontin from bone marrow-derived mesenchymal stromal cells improve muscle regeneration
Source: Sci Rep. 2017 Jun 12;7:3305. doi: 10.1038/s41598-017-02928-1 (PMC5468354; doi:10.1038/s41598-017-02928-1)
Supplement: Supplementary file 1 — suplementary methods, suplementary figure legend [file 41598_2017_2928_MOESM1_ESM.pdf]

Methods

Isolation and expansion of Bm-MSCs. We obtained Bm-MSCs from two dko mice, female and male, when they were 6–8 weeks old. Mice were killed by using CO_2,after which the femurs and tibias were removed, cleaned of all connective tissue, and plated on ice in 2 ml of complete isolation medium (CIM). CIM consisted of RPMI-1640 (Invitrogen, Carlsbad, CA) supplemented with 9% FBS (Cell Culture Bioscience, Tokyo, Japan), 9% horse serum (HS; Hyclone Laboratories, Logan, UT), 100 U/ml penicillin (Invitrogen), 100 μg/ml streptomycin (Invitrogen), and 12 μM L-glutamine (Invitrogen). We clipped the ends of each tibia and femur to expose the marrow. We inserted the bones into adapted centrifuge tubes and centrifuged them for 1 minute at 400 × *g* to collect the marrow. We suspended the pellets in 3 ml of CIM by using a 21-gauge syringe followed by filtration through a 70-μm nylon mesh filter. Cells from long bones were plated in 40 ml of CIM in a 175-cm^2 flask. After 24 hours, nonadherent cells were removed by washing them with PBS, and 30 ml of fresh CIM was added. Adherent cells (passage 0) were washed, and fresh CIM was added every 3–4 days for 4 weeks. Cells were then washed with PBS and lifted by incubation in 3 ml of 0.25% trypsin/1 mM EDTA for 2 minutes at 37°C. Cells that did not lift in 2 minutes were discarded. Trypsin was neutralized by adding 8 ml of CIM, and all cells (passage 1) from one flask were replated in 25 ml of CIM in a 175-cm^2

flask. CIM was replaced every 3–4 days. After 2 weeks, cells were lifted by incubation with 0.25% trypsin/1 mM EDTA for 2 minutes at 37°C. These cells (passage 2) were then expanded by plating at 50 cells/cm^2 in complete expansion medium (CEM) consisting of Iscove's modified Dulbecco medium (IMDM; Invitrogen) supplemented with 9% FCS, 9% HS, 100 U/ml penicillin (Invitrogen), 100 µg/ml streptomycin (Invitrogen), and 12 µM L-glutamine (Invitrogen). CEM was replaced every 3–4 days. After 10 days, these cells (passage 3) were lifted by incubation with 0.25% trypsin/1 mM EDTA for 2 minutes at 37°C. Passage 3 cells were either frozen or expanded further by plating at 50 cells/cm^2 and incubating them in CEM. For freezing, cells were resuspended at 3.0×10^5 cells/ml in 5% DMSO and 95% FBS, frozen at −80°C for 24 hours, and then stored in liquid nitrogen. To recover frozen cells, a vial was quickly thawed to 37°C, and cells were plated in a 175-cm^2 flask in CEM and incubated for 2 days. After 2 days, the flask was washed with PBS, and cells were lifted by incubation with 0.25% trypsin/1 mM EDTA for 2 minutes at 37°C, plated at 50 cells/cm^2 in CEM, and incubated for 12 days, with the medium changed every 3–4 days. The same conditions were used for successive passages.

Differentiation of Bm-MSCs. We plated cells at 50 cells/cm^2 in 6-well plates and

incubated them in CEM for 10 days. For osteogenesis, cultures were then incubated in IMDM that was supplemented with 10% FCS, 10% HS, 100 U/ml penicillin, 100 μg/ml streptomycin, 12 mM L-glutamine, 20 mM β-glycerol phosphate (Sigma, St. Louis, MO), 50 ng/ml thyroxine (Sigma), 1 nM dexamethasone (Sigma), and 0.5 μM ascorbate-2-phosphate (Sigma). The medium was changed two times per week for 3 weeks. Cells were fixed in 10% formalin for 20 minutes at room temperature and were stained with Alizarin Red, pH 4.1 (Sigma), for 20 minutes at room temperature. For adipogenesis, cultures were incubated in IMDM that was supplemented with 10% FCS, 10% HS, 100 U/ml penicillin, 100 μg/ml streptomycin, 12 mM L-glutamine, 5 μg/ml insulin (Sigma), 50 μM indomethacin (Sigma), 1×10^{-6} M dexamethasone, and 0.5 μM 3-isobutyl-1-methylxanthine (Sigma). The medium was changed two times per week for 3 weeks. Cells were fixed in 10% formalin for 20 minutes at room temperature and were then stained with 0.5% Oil Red O (Sigma) in methanol (Wako, Osaka, Japan) for 20 minutes at room temperature.

FACS analysis of Bm-MSCs. We used the Mouse Multipotent Mesenchymal Stromal Cell Marker Antibody Panel containing the following antibodies: anti-Sca-1 (clone 177228, catalog FAB1226P; R&D Systems, Minneapolis, MN); anti-CD105 (clone

209701, catalog FAB1320P; R&D Systems); anti-CD106 (clone 112734, catalog FAB6432P; R&D Systems); anti-CD44 (clone IM7, catalog clone IM7; R&D Systems); anti-CD11b (clone M1/70, catalog MAB1124; R&D Systems); anti-CD45 (clone 30-F11, catalog FAB114F-025; R&D Systems); isotype control rat IgG_{2A} (clone MAB006; R&D Systems); isotype control rat IgG_{2B} (clone MAB0061; R&D Systems); and secondary developing reagents (R&D Systems). We followed the manufacturer's instructions. In brief, Bm-MSCs cultured in CEM were lifted with trypsin/EDTA and resuspended in Flow Cytometry Staining Buffer at a concentration of 1.0×10^6 cells/ml. Cells were added with each antibody or with isotype-matched control antibody (10 μl in 100 μl), followed by incubation for 30 minutes at 4°C. After incubation, cells were washed twice and resuspended in the same buffer. Resuspended cells were added to a secondary developing reagent according to the manufacturer's instruction, followed by incubation for 30 minutes at 4°C in the dark. Cells were washed twice and resuspended in the same buffer for flow cytometry.

Isolation of single myofibers and their culture[45]. We explanted single myofibers for culture from the left and right calf muscles (soleus and gastrocnemius) from C57BL/10 (wild-type) mice. To analyze myofiber appearance and the number of

satellite cells (SCs) on myofibers, single fibers were isolated from dko mice and dko/MSC mice. For myofiber isolation and culture, culture medium must contain 110 mg/ml sodium pyruvate so that fibers survive. Mice were killed by using cervical dislocation, and muscles were removed immediately by microdissection, with care being taken to handle muscles only by their tendons to minimize damage to the fibers. The removed muscles were rinsed with PBS, were put into a 25-ml centrifuge tube with a triple seal cap (Iwaki, Tokyo, Japan) containing 5 ml of fresh filter-sterilized 0.2% (w/v) type I collagenase (Worthington Biochemical, Lakewood, NJ) in DMEM plus GlutaMAX-I (Gibco, Grand Island, NY) supplemented with 2 mM L-glutamine (Gibco), and were incubated in a CO_2 incubator at 37°C for 1.5–2 hours. After the muscles were digested, samples were transferred, with 5-3/4-inch Pasteur pipettes (Iwaki), to the first of a series of 50 mm × 20.3 mm plastic Petri dishes (Thermo Fisher Scientific) containing 20 ml of DMEM. To prevent fibers from sticking to the Petri dishes and the pipettes, the former were prerinsed and the latter were preflushed with 5% BSA (Sigma) in PBS. We used a transilluminating stereo dissecting microscope to liberate single muscle fibers by repeated flushes of medium with the 5-3/4-inch Pasteur pipettes. After 20–30 intact, viable single muscle fibers were separated, the muscle bulk was transferred to a fresh Petri dish, and the previous

dish, containing separated fibers and “debris” (hypercontracted fibers, fiber pieces, fiber clusters, and collagen wisps), was incubated in a humid environment at 37°C and 5% CO_2. We repeated the fiber separation cycle until we had dissociated sufficient numbers of fibers from the muscles. Intact, viable muscle fibers were then separated from debris by transferring them with a normal Pasteur pipette to a fresh Petri dish, in which they were incubated until plating. Before plating, wells of 6-well tissue culture plates were briefly rinsed with 50% Matrigel (Becton, Dickinson, Franklin Lakes, NJ), which was applied ice-cold to prevent premature gelation, and plates were then incubated for 30 minutes at 37°C. Individual muscle fibers were removed from suspension with a 9-inch Pasteur pipette (Iwaki), and each fiber was placed in the center of a well with 2 ml of plating medium. Plating medium consisted of DMEM plus GlutaMAX-I supplemented with 2 mM L-glutamine and 10% HS. During the first 3 days, the fibers were cultured in plating medium. On the fourth day, culture medium was changed to proliferation medium, which consisted of DMEM plus GlutaMAX-I supplemented with 2 mM L-glutamine, 10% HS, 20% FBS, and 1% (v/v) chick embryo extract (United States Biological, Salem, MA). The medium was changed every 3 days. One day before coculture, Bm-MSCs (1.0×10^5 cells) were added to each 30-mm cell culture plate insert, Preset VECELL (Vessel, Kitakyushu, Japan), and were

maintained in CEM. When single fibers were placed in 6-well culture plates, they were immediately covered by culture inserts containing Bm-MSCs.

CXCL12, OPN, and CSF-1 cDNA amplification from Bm-MSCs via PCR. Total RNA purification from Bm-MSCs and first-strand cDNA synthesis were achieved. Full-length open reading frame cDNAs for CXCL12, OPN, and CSF-1 were amplified by using Ampdirect Plus (Shimazu, Kyoto, Japan) and NovaTaq DNA Polymerase (Novagen, Darmstadt, Germany) according to the manufacturers' instructions. Primer sequences were the following:

Kozak-CXCL12-F: 5'-CGGCCGCCACCATGGACGCCAAGGTCGT-3',

Kozak-CXCL12-R: 5'-TCGCGAGTCCTTTGGGCTGTTGTGC-3',

Kozak-OPN-F: 5'-CGGCCGCCACCATGAGGCTGCAGTTCTCC-3',

Kozak-OPN-R: 5'-TCGCGAGGAACTGTGTTTTTGCCTCTT-3',

Kozak-CSF-1-F: 5'-CGGCCGCCACCATGACCGCGCGGGGCGCC-3',

Kozak-CSF-1-R: 5'-TCGCGAGTGTGCCCAGCATAGAATCC-3'.

After the reactions, products were separated on 1.0% agarose gel.

CXCL12 expression vector construction and CXCL12 preparation. As we

mentioned above, the forward primer contained a Kozak consensus sequence for efficient translation. The amplified product was first cloned into the pCR-Blunt II-TOPO (Thermo Fisher Scientific). After we confirmed the sequence, CXCL12 cDNA carrying the Kozak sequence was re-cloned into pcDNA (pRc/RSV; Invitrogen) mammalian expression vector and was named pcDNA-CXCL12. The expression vector was purified from the large-scale culture by means of the Plasmid Giga Kit (QIAGEN, Tokyo, Japan).

pcDNA-CXCL12 was transfected into HEK293 cells cultured in 10% FCS-containing DMEM by the conventional calcium phosphate precipitation method. After precipitation, cells were cultured overnight, and then they were washed thoroughly with PBS to remove precipitates and expression vector, after which they were cultured as usual in 10% FCS/DMEM for 1 more day to wait for efficient CXCL12 transcription. On the third day after transfection, to remove FCS from the culture, cells were washed repeatedly with PBS followed by changing the culture medium to a serum-free medium. After 2 more days of culture, we collected the medium that we expected contained CXCL12 secreted from transfected HEK293 cells.

We quantified the mouse CXCL12 concentration by using ELISA for mouse-specific CXCL12. We also quantified human CXCL12 from HEK293 cells, to see whether it was

present, by using human-specific ELISA, but human CXCL12 was below the detectable concentration.

As we mentioned above, we prepared mouse-specific CXCL12 under serum-free conditions. When we used CXCL12 *in vivo* and *in vitro*, supernatant of serum-free medium collected from the mock-transfected HEK293 cells was used as a negative control.

CXCL12 concentration in culture medium of Bm-MSCs as determined by ELISA.

Bm-MSCs were expanded by plating at 50 cells/cm^2 in CEM consisting of IMDM (Invitrogen) supplemented with 9% FCS, 9% HS, 100 U/ml penicillin (Invitrogen), 100 μg/ml streptomycin (Invitrogen), and 12 μM L-glutamine (Invitrogen). After 2 days of culture, we removed FCS from the culture and washed the cells repeatedly with PBS. We changed the culture medium to a serum-free medium. After 2 more days of culture, we collected the medium and quantified the mouse CXCL12 concentration by using ELISA (CXCL12, Mouse, ELISA Kit, Quantikine; R&D Systems).

Muscle SC isolation and culture (Supplementary Fig. S5). We isolated muscle SCs as previously described[46]. We removed limb muscles from mice and minced them. We

placed these minced muscles into a 25-ml centrifuge tube with a triple seal cap (Iwaki) containing 5 ml of fresh, filter-sterilized 0.5% (w/v) type I collagenase (Worthington Biochemical) in DMEM plus GlutaMAX-I (Gibco) supplemented with 2 mM L-glutamine (Gibco), followed by incubation in a 5% CO_2 incubator at 37°C for 1.5-2 hours. After this digestion step, we passed the remaining cell suspension through sequential 40-μm filters (BD Biosciences, Franklin Lakes, NJ) to generate single-cell suspensions. We first incubated cells with biotinylated antibodies that reacted with CD45 (clone 30F11, catalog 553078; BD Biosciences), CD11b (clone M1/70, catalog 553309; BD Biosciences), CD31 (clone 390, catalog 13-0311-82; BD Biosciences), and Sca-1 (clone E13-161.7, catalog 553334; BD Biosciences). We incubated the cells with streptavidin magnetic beads (Miltenyi Biotec, Bergisch Gladbach, Germany) and depleted biotin-positive cells with a selection column (Miltenyi Biotec). We then sorted biotin-negative cells on an FACS Aria II cell sorter (Supplementary Fig. S5a) by using FACSDiva software (BD Biosciences). We used the following antibodies: anti-integrin-α7 (clone 334908, catalog FAB3518P; R&D Systems), anti-CD34 (clone 700011, catalog FAB6518G; R&D Systems), anti-CXCR4 (clone 247506, catalog FAB21651P; R&D Systems), isotype control Rat IgG_{2A} (clone 141945, catalog IC013P; R&D Systems), and isotype control Rat IgG_{2B} (clone 54447,

catalog IC006G; R&D Systems). We enriched SCs by gating viable cells for cells negative for the lineage marker panel (CD45, CD31, CD11b, and Sca-1) and positive for CD34 and integrin-α7. We generated and analyzed flow cytometry scatter plots by using FlowJo v8.7 (Tree Star, Ashland, USA). We plated the SCs in 6-well tissue culture plates with 50% Matrigel (Becton, Dickinson); cultured them in medium that consisted of DMEM plus GlutaMAX-I supplemented with 2 mM L-glutamine, 10% HS, and 20% FBS; and incubated them in a 5% CO_2 incubator at 37°C. We changed the medium every 3 days. We cultured SCs for 3 days and immunostained these cells (Supplementary Fig. S5b). We confirmed that SCs expressed PAX7. Furthermore, we cultured SCs for 7 days and confirmed that myotubes were formed (Supplementary Fig. S5c).

Kinex Antibody Microarray. We isolated SCs by using FACS, harvested 1.0 × 10^5 cells, and plated them in 6-well tissue culture plates with 50% Matrigel (Becton, Dickinson). We cultured the cells in medium that consisted of DMEM plus GlutaMAX-I supplemented with 2 mM L-glutamine, 10% HS, and 20% FBS and incubated them in a 5% CO_2 incubator at 37°C for 6 days. We changed this medium and added CXCL12 every 5 days. On day 6, we performed the protein array studies by using

Kinex Antibody Microarray Services (Kinexus Bioinformatics, Vancouver, B.C., Canada).

Supplementary References

45. Rosenblatt, J. D., Lunt, A. I., Parry, D. J. & Partridge, T. A. Culturing satellite cells from living single muscle fiber explants. *In Vitro Cell. Dev. Biol. Anim.* **31**, 773-779 (1995).

46. Sacco, A., Doyonnas, R., Kraft, P., Vitorovic, S. & Blau, H. M. Self-renewal and expansion of single transplanted muscle stem cells. *Nature* **456**, 502-506 (2008).

Supplementary Figure S1. Bm-MSCs have null mutations in both *dystrophin* and *utrophin* genes. (**a**) Status of the *dystrophin* gene. Bm-MSCs used in this experiment had only an *mdx* mutation in the *dystrophin* gene, because of successful amplification by a mutation-specific primer pair. (**b**) Status of the *utrophin* gene. Bm-MSCs had only a null mutation in the *utrophin* gene: +/+: wild type, -/+: mutant allele with wild-type allele, -/-: null mutation.

Supplementary Figure S2. Characterization of Bm-MSCs. (**a**) We harvested and

cultured cells from femoral and tibial bone marrow in *dystrophin/utrophin* dko mice by using Prockop's protocol. The nonsense mutation in the *dystrophin* gene was derived from the *mdx* mouse, and the phosphoglycerate kinase-neomycin insertion into exon 7 of the *utrophin* gene was derived from the gene-targeted mouse. These cultured cells were the Bm-MSCs. (**b**) Cells expressed the mesenchymal cell surface markers Sca-1, CD44, CD105, and CD106, but not CD45 or CD11b. (**c**) After four passages, the cultured cells successfully differentiated into osteoblasts and adipocytes. Scale bars: 100 μm.

Supplementary Figure S3. ELISA showed that CXCL12 was expressed in the culture medium of Bm-MSCs.

Supplementary Figure S4. p-STAT3 expression was not suppressed in the other cell lines: COS7, C2C12, and RD.

Supplementary Figure S5. FACS analysis of muscle SCs and immunostaining. (**a**) We used FACS to isolate muscle SCs, which were $CD34^+$ and integrin-$\alpha 7^+$. About 70% of these cells were also $CXCR4^+$. (**b**) We incubated muscle SCs for 4 days, followed by

immunofluorescence staining, which allowed identification of SCs, as this representative image shows (nucleus, red, arrowheads; PAX7, green, arrows). Scale bar: 100 μm. (**c**) We incubated muscle SCs for 7 days and observed them with the All-in-One Fluorescence Microscope BZ-9000 (Keyence). Scale bar: 100 μm.

Supplementary Table S1. Protein array studies performed by using Kinex Antibody Microarray Services. STAT3 was not changed.

Supplementary movie. Despite defects in the *dystrophin* and *utrophin* genes, Bm-MSC-transplanted dko mice moved vigorously. Two of five mice were Bm-MSC-transplanted dko mice. Those mice are easily distinguishable from the untreated dko mice.
