# Supplementary material for: CXCL12 and osteopontin from bone marrow-derived mesenchymal stromal cells improve muscle regeneration
Source: Sci Rep. 2017 Jun 12;7:3305. doi: 10.1038/s41598-017-02928-1 (PMC5468354; doi:10.1038/s41598-017-02928-1)

CXCL12 and osteopontin from bone marrow-derived mesenchymal stromal cells improve muscle regeneration

Yasushi Maeda,[1*] Yasuhiro Yonemochi,[2*] Yuki Nakajyo,[2] Hideaki Hidaka,[2] Tokunori Ikeda,[3] and Yukio Ando[2]

[1]Department of Neurology, National Hospital Organization Kumamoto Saishunso National Hospital, Kumamoto, Japan. [2]Department of Neurology, Graduate School of Medical Sciences, Kumamoto University, Kumamoto, Japan. [3]Department of Clinical Research Center, Faculty of Life Sciences, Kumamoto University, Kumamoto, Japan.

Supplementary Figure S1

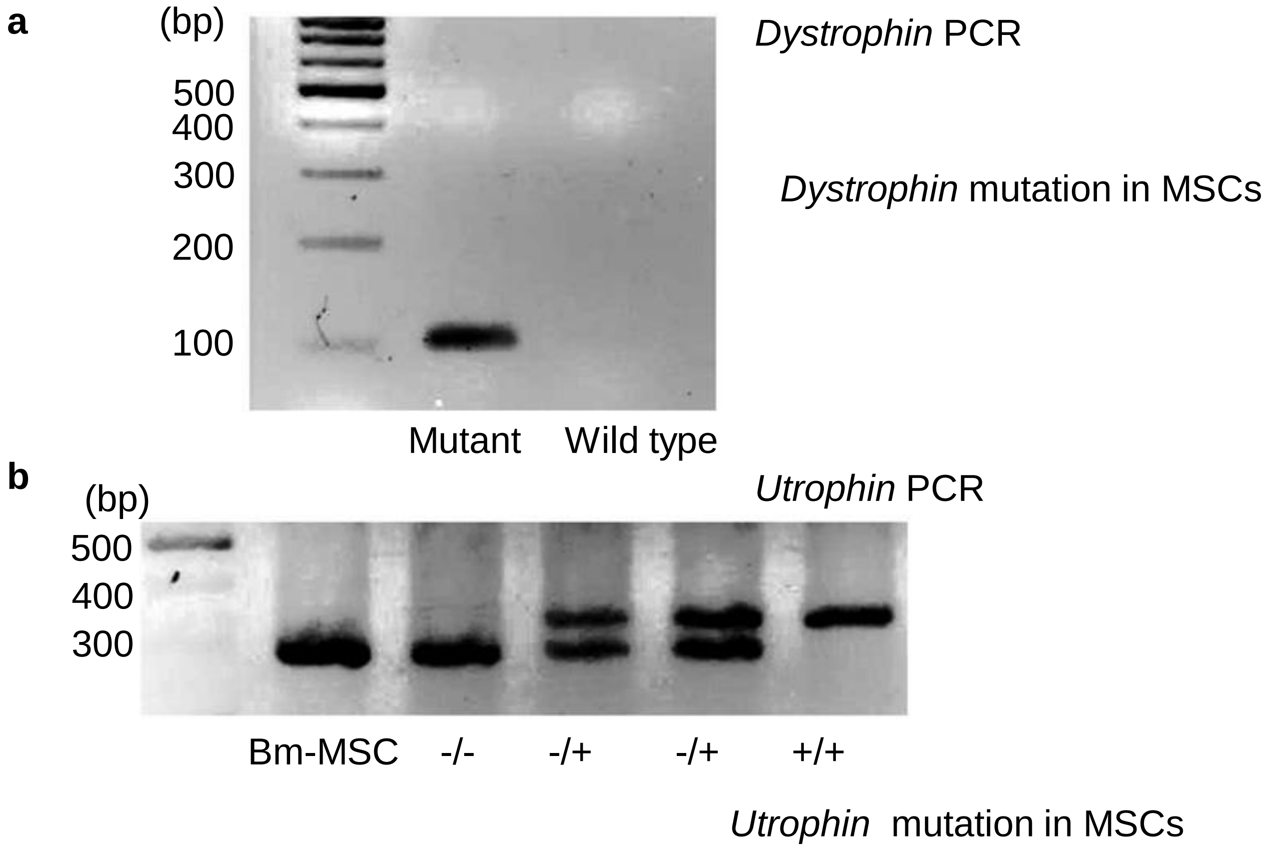

Supplementary Figure S2

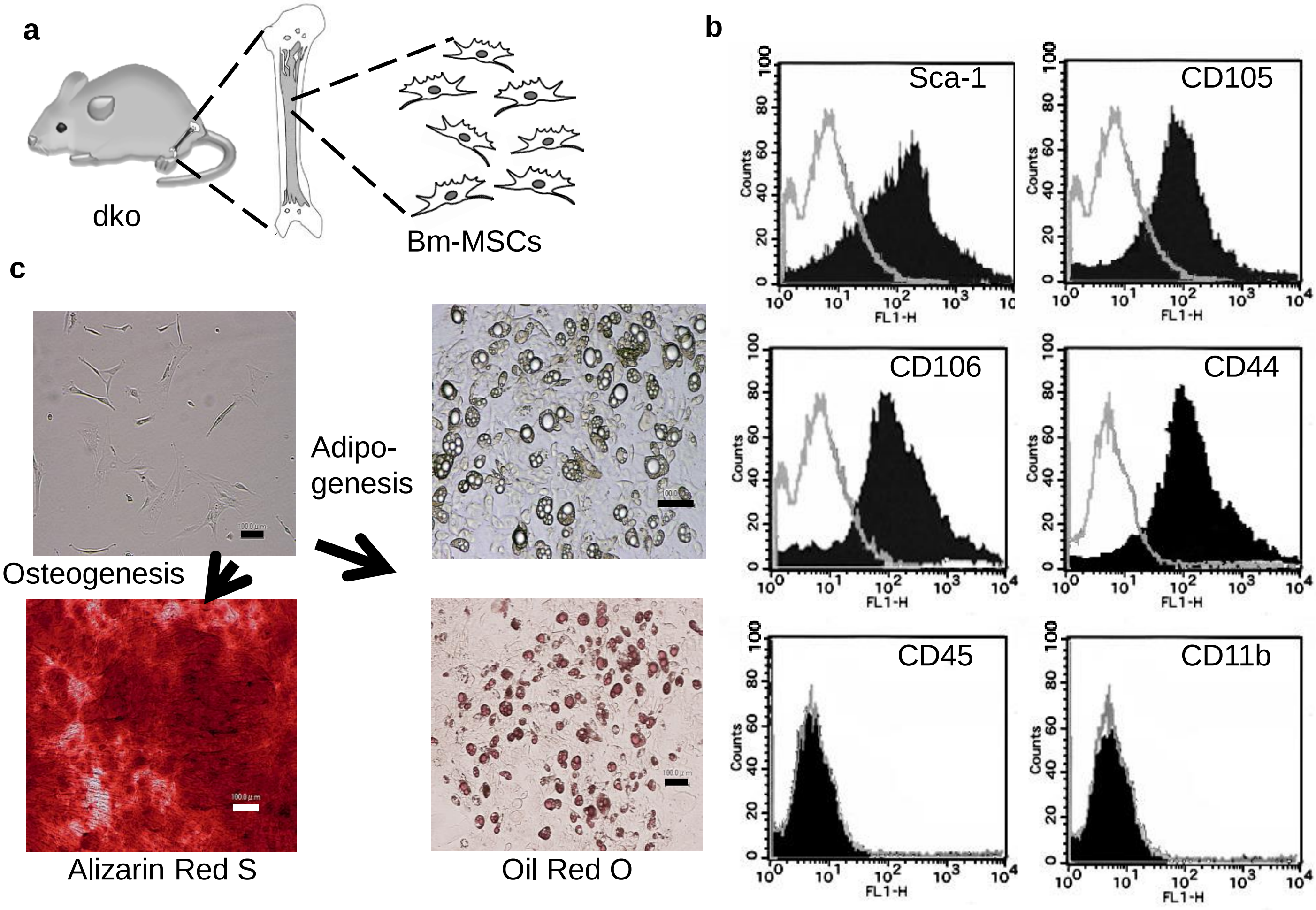

Supplementary Figure S3

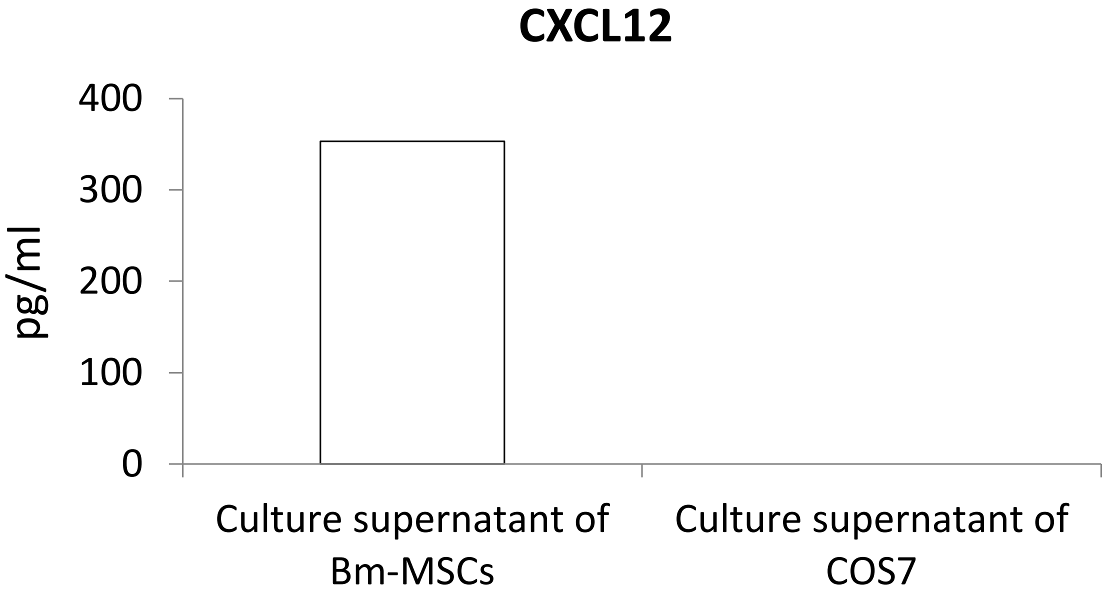

Supplementary Figure S4

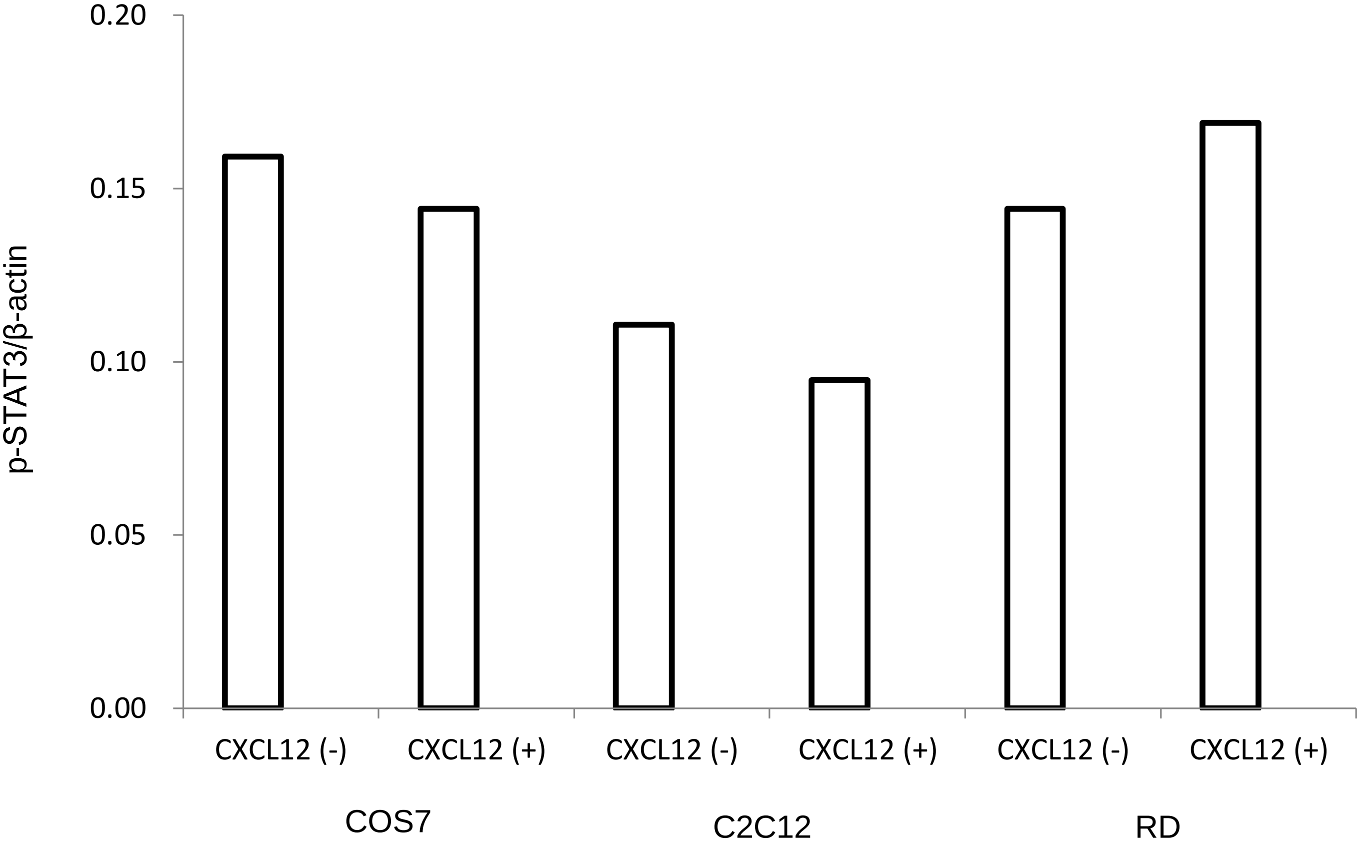

Supplementary Figure S5

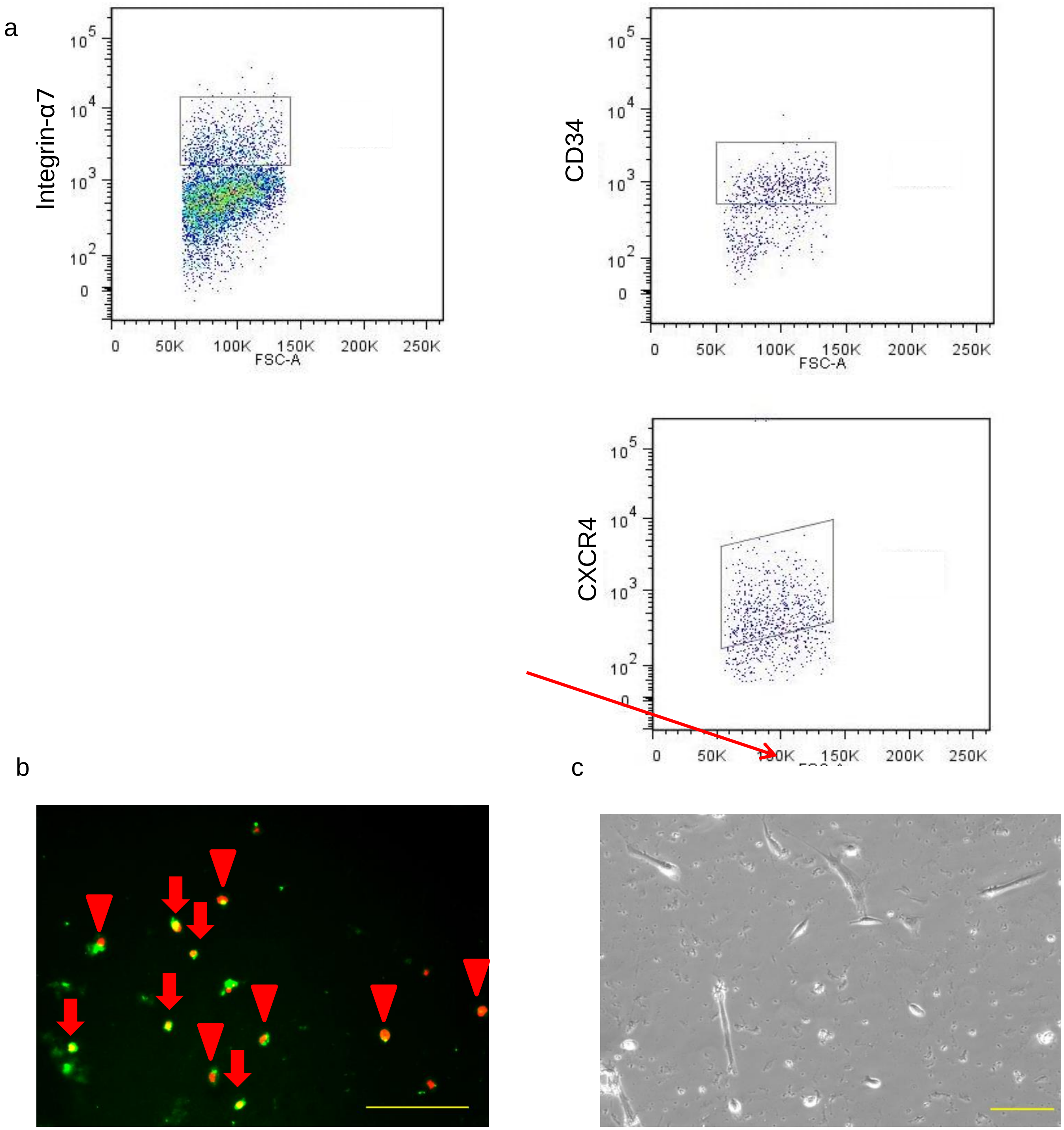

Supplement: Supplementary file 2 — suplementary figure S1–S5 [file 41598_2017_2928_MOESM2_ESM.pdf]
