# Supplementary material for: CXCL12 and osteopontin from bone marrow-derived mesenchymal stromal cells improve muscle regeneration
Source: Sci Rep. 2017 Jun 12;7:3305. doi: 10.1038/s41598-017-02928-1 (PMC5468354; doi:10.1038/s41598-017-02928-1)
Supplement: Supplementary file 3 — suplemental table S1 [file 41598_2017_2928_MOESM3_ESM.pdf]

CXCL12 and osteopontin from bone marrow-derived mesenchymal stromal cells improve muscle regeneration

Yasushi Maeda,[1*] Yasuhiro Yonemochi,[2*] Yuki Nakajyo,[2] Hideaki Hidaka,[2] Tokunori Ikeda,[3] and Yukio Ando[2]

[1]Department of Neurology, National Hospital Organization Kumamoto Saishunso National Hospital, Kumamoto, Japan. [2]Department of Neurology, Graduate School of Medical Sciences, Kumamoto University, Kumamoto, Japan. [3]Department of Clinical Research Center, Faculty of Life Sciences, Kumamoto University, Kumamoto, Japan.

Supplementary Table S1.

Target protein name	Flag- satellite cell mock+	Globally normalized – satellite cell mock+	% Error range satellite cell mock+	Log_2 (intensity corrected) – satellite cell mock+	Flag- satellite cell CXCL12+	Globally normalized - satellite cell CXCL12+	% Error range satellite cell CXCL12+	Log_2 (intensity corrected) – satellite cell CXCL12+	% CFC (satellite cell CXCL12+ from satellite cell mock+)	Best leads	Z-score (satellite cell mock+)	Z-score (satellite cell CXCL12+)	Z-score difference (satellite cell CXCL12+ – satellite cell mock+)	Z-ratio (satellite cell CXCL12+, satellite cell mock+)
STAT3	0, 0	1668	23	8.29	0, 0	2063	22	8.27	24		-0.08	0.06	0.14	0.86

Supplementary Table S1. Protein array studies performed by using Kinex Antibody Microarray Services. STAT3 was not changed.
